# Draft Genome Sequence of *Streptomyces* sp. Isolate H28 from the Meycauayan River, Philippines

**DOI:** 10.1128/MRA.01269-20

**Published:** 2021-01-07

**Authors:** Aroin B. Verdera, Andrew D. Montecillo, Marie Christine M. Obusan

**Affiliations:** a Microbial Ecology of Terrestrial and Aquatic Systems Laboratory, Institute of Biology, University of the Philippines Diliman, Quezon City, Philippines; b Institute of Biological Sciences, University of the Philippines Los Baños, Laguna, Philippines; c Natural Sciences Research Institute, University of the Philippines Diliman, Quezon City, Philippines; Georgia Institute of Technology

## Abstract

In this paper, we report the draft genome sequence of *Streptomyces* sp. isolate H28, isolated from sediments of the Meycauayan River in the Philippines. This species exhibits production of melanin as well as the ability to utilize and degrade both high-density polyethylene (HDPE) and low-density polyethylene (LDPE).

## Abstract (precis)

In this paper, we report the draft genome sequence of *Streptomyces* sp. isolate H28, isolated from the sediments of Meycauayan River, Philippines. This species exhibits production of melanin as well as the ability to utilize and degrade both high-density polyethylene (HDPE) and low-density polyethylene (LDPE).

## ANNOUNCEMENT

A *Streptomyces* species called isolate H28 was isolated from sediments of the Meycauayan River, a segment of the Marilao-Meycauayan-Obando River System (MMORS) in the Philippines (14°44′14.29″N, 120°57′26.11″E) that receives effluents from gold smelters, a public market, and households.

Pure culture of isolate H28 was grown and maintained in soyabean-casein digest agar (15 g tryptone, 5 g soya peptone, 5 g NaCl, and 15 g agar per liter). The genomic DNA of isolate H28 was extracted using a KingFisher cell and tissue DNA kit (Thermo Fisher Scientific, USA) according to the manufacturer’s protocol. A Nextera XT library prep kit was used for the preparation of the sequencing library, and Illumina MiSeq 2 × 300-bp reads were used for sequencing. A total of 1,390,602 paired-end reads comprising 379,629,799 bases were obtained. The Q-score heat map shows that the first ∼300 bp of read 1 sequences were above Q30, and the first ∼220 bp of read 2 sequences were above Q30.

Quality control of raw reads was done using the program fastp v0.19.8 (1) using the following parameters: -3, -q 20, and -T 114. After filtering, there were a total of 999,998 reads and 180.917 million bases. Of these, 167.337 million are Q20 bases (92.49%) and 143.117 million are Q30 bases (79.11%). Genomic assembly was done using SPAdes v3.13 (2) using the default parameters and the --careful option. The assessment of the quality of the assembly was done using QUAST (3). The assembly, with an estimated genome coverage of 26×, comprises 1,181 contigs (1,180 scaffolds) with a total of 6,697,207 bases. The largest contig and scaffold is 58,123 bp long. It has a GC content of 72.73%, and the *N*_50_ and *L*_50_ lengths are 9,931 bp and 207 bp, respectively.

Using the Microbial Genomes Atlas (MiGA) v0.7.15.2 (4), the closest taxonomic relative was found to be Streptomyces viridosporus T7A NZ (GenBank accession number CP023700, GenBank assembly accession number GCA_008704515.1) (*P* value, 0.419) with 87.87% average nucleotide identity (ANI) and a 69.37% fraction of proteins shared. Isolate H28 likely belongs to a species not represented in the database (*P* value, 0.0043). Using MiGA, the assembly was found to have 105 out of the 106 (99.1%) essential genes found in *Bacteria* and *Archaea* in addition to 6,558 predicted proteins with an average length of 296.44 amino acids.

A total of 7,103 new features were predicted via the Rapid Annotations using Subsystems Technology (RAST) tool kit (5). Of these, 319 are noncoding and 6,784 are coding genes. Overall, the coding genes were identified to have 3,352 distinct functions. Genes coding for proteins that are associated with plastic degradation, such as esterase, lipase, P450 hydroxylase, multicopper oxidase, and hydroxylase were found. Genes coding for putative tyrosinase and tyrosinase cofactor that are associated with melanin production were also found.

To identify secondary metabolite gene clusters, antiSMASH v5.1.2 (6) was used. The genome was found to have 30 biosynthetic gene clusters coding for a total of 13 secondary metabolite types, including melanin. The following 30 secondary metabolite regions were identified: lanthipeptide (1 region), terpene (8 regions), siderophore (3 regions), melanin (3 regions), nonribosomal peptides (NRPS) (6 regions), ectoine (1 region), butyrolactone (1 region), bacteriocin (2 regions), type II polyketide synthase (T2PKS) (1 region), furan (1 region), ladderane (1 region), indole (1 region), and type I polyketide synthase (T1PKS) (1 region).

All software programs were run using the default parameters unless mentioned otherwise.

### Data availability.

This whole-genome sequencing project has been deposited at GenBank under the accession numbers JACYXY010000001 to JACYXY010001180, BioProject number PRJNA665970, BioSample number SAMN16275722, and Sequencing Read Archive (SRA) number SRR13061778.
